# Supplementary figures and images for: Integrative Analysis and Experimental Validation of Competing Endogenous RNAs in Obstructive Sleep Apnea
Source: Biomolecules. 2023 Apr 1;13(4):639. doi: 10.3390/biom13040639 (PMC10135462; doi:10.3390/biom13040639)

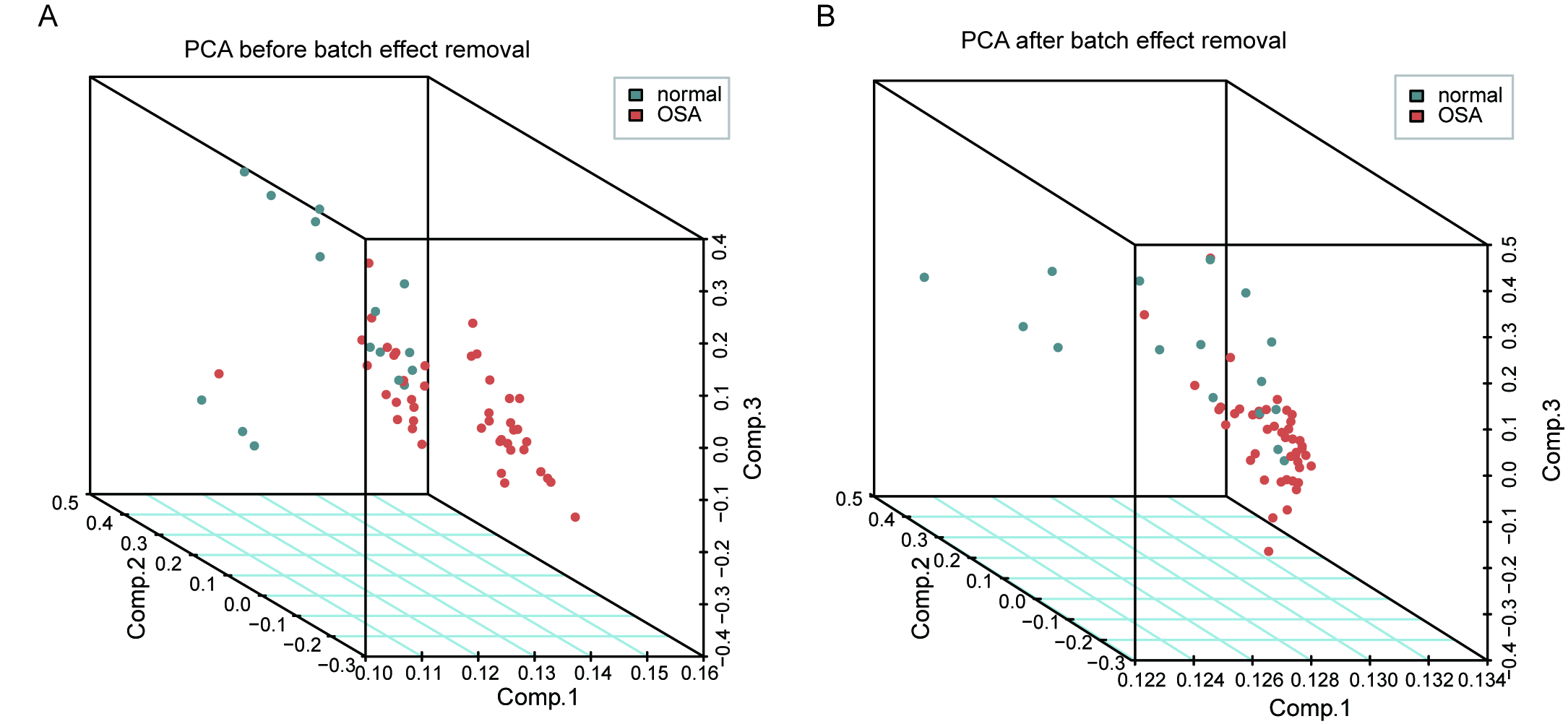

Supplement: Supplementary file 1 [file biomolecules-13-00639-s001.zip › Supplementary information, Figure S1.tif]

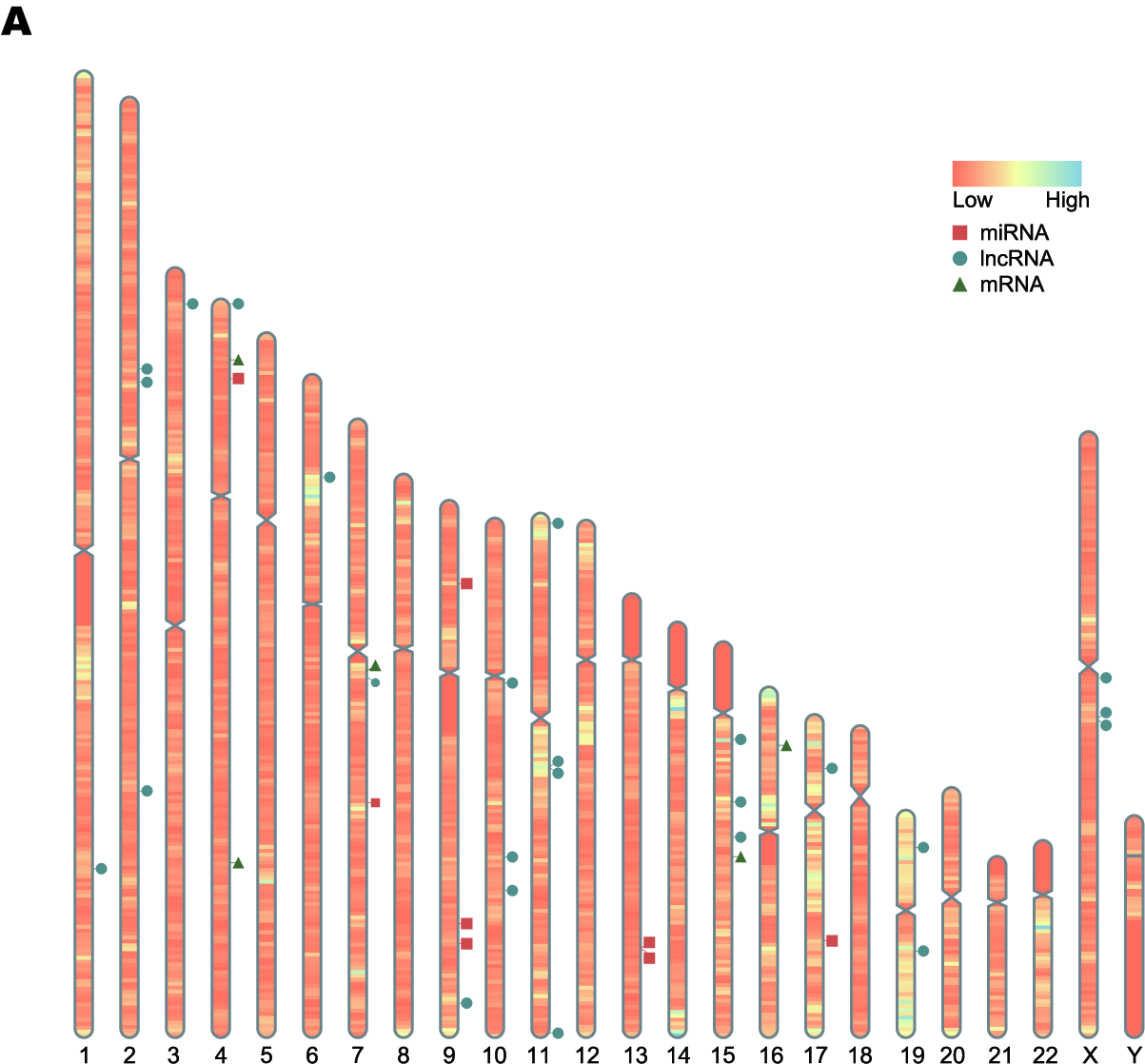

Supplement: Supplementary file 1 [file biomolecules-13-00639-s001.zip › Supplementary information, Figure S2.tif]

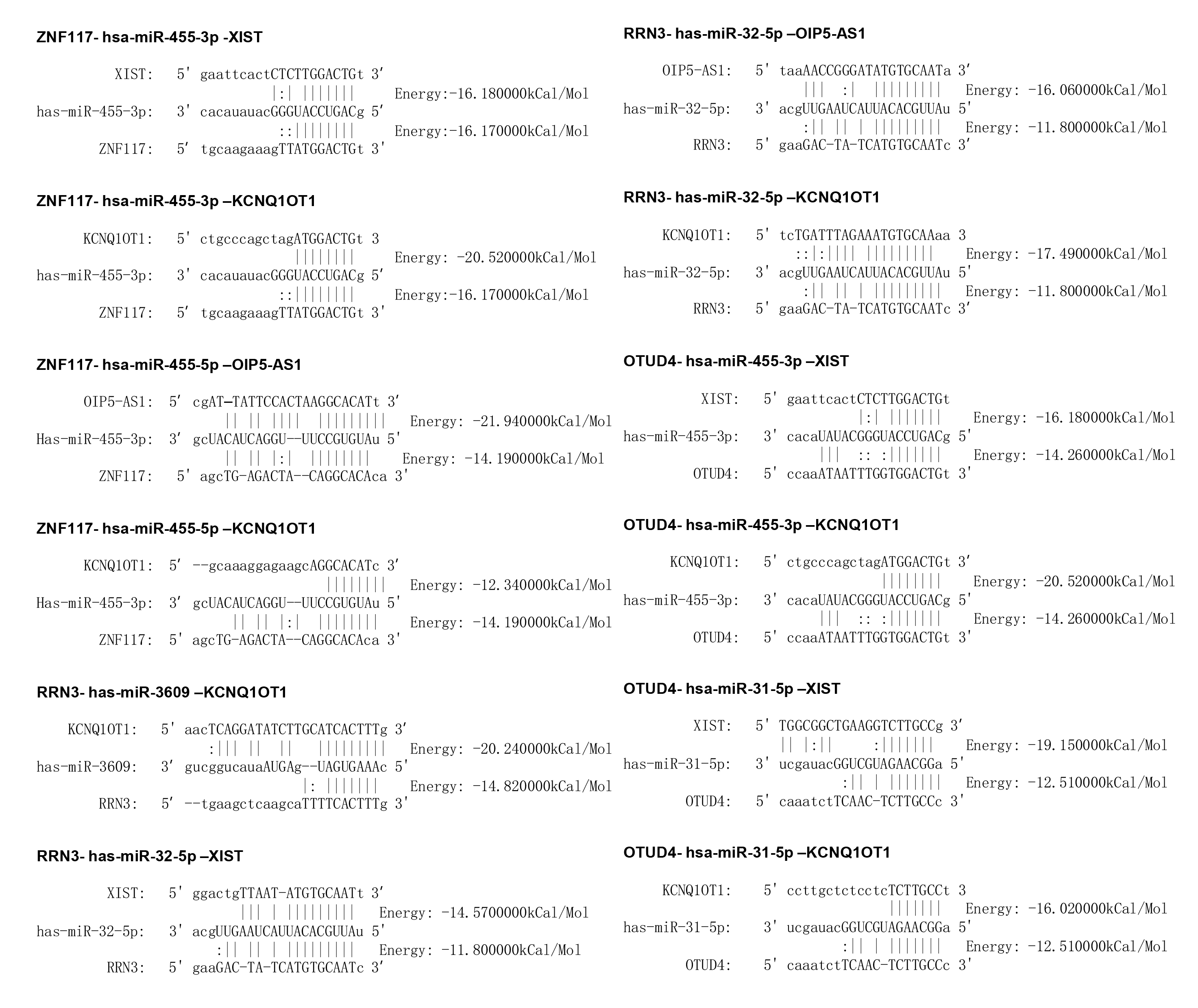

Supplement: Supplementary file 1 [file biomolecules-13-00639-s001.zip › Supplementary information, Figure S3.tif]
